# Supplementary material for: The grain yield modulator miR156 regulates seed dormancy through the gibberellin pathway in rice
Source: Nat Commun. 2019 Aug 23;10:3822. doi: 10.1038/s41467-019-11830-5 (PMC6707268; doi:10.1038/s41467-019-11830-5)
Supplement: Supplementary file 1 — Supplementary Information [file 41467_2019_11830_MOESM1_ESM.pdf]

Supplementary Information

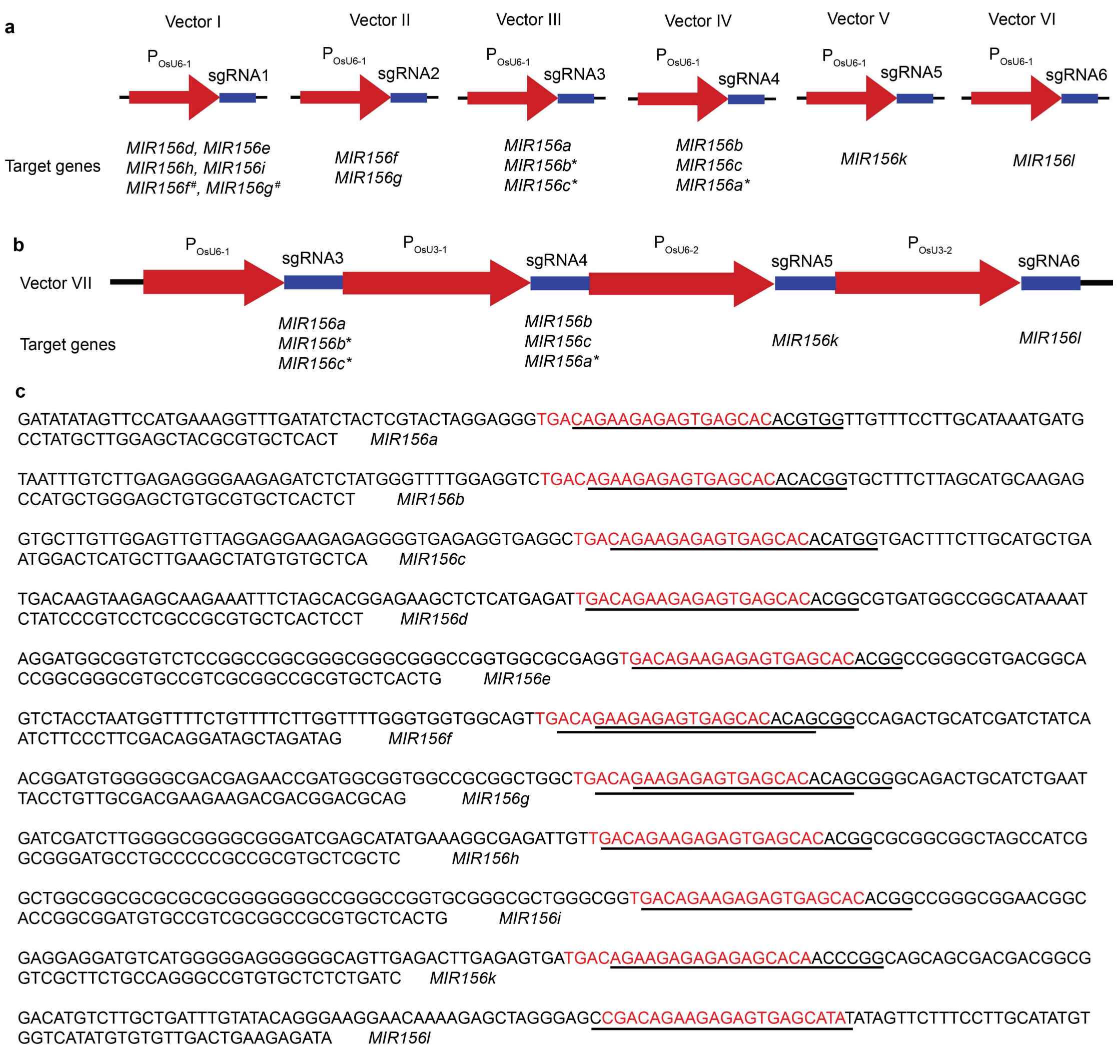

Supplementary Figure 1 Strategy for rice *MIR156* gene editing. (**a**) Single sgRNA-expression vectors for *MIR156* gene editing. #, target with CAG as the protospacer-adjacent motif (PAM); *, target with a mismatch to the sgRNA directly 5’ of the PAM; red arrows, promoters; violet-blue boxes, sgRNA-expressing sequences. (**b**) Multiplex gene editing for group II *MIR156s*. *, target with a mismatch to the sgRNA directly 5’ of the PAM; red arrows, promoters; violet-blue boxes, sgRNA-expressing sequences. (**c**) Cas9 target sites for *MIR156* gene editing. The sequences with red color correspond to the mature miR156 sequences. The underlined sequences are the target sites for Cas9.

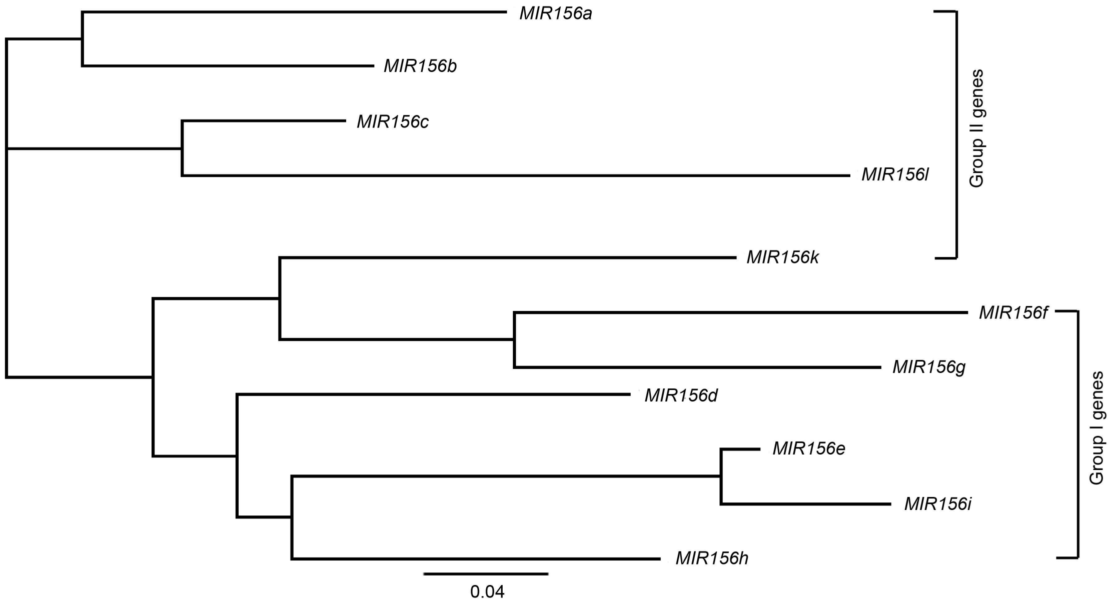

Supplementary Figure 2 Phylogenetic tree of rice *MIR156s*. The phylogenetic tree was constructed with 100-bp genomic sequences (20-bp upstream sequences + sequences corresponding to mature miR156 sequences + downstream sequences).

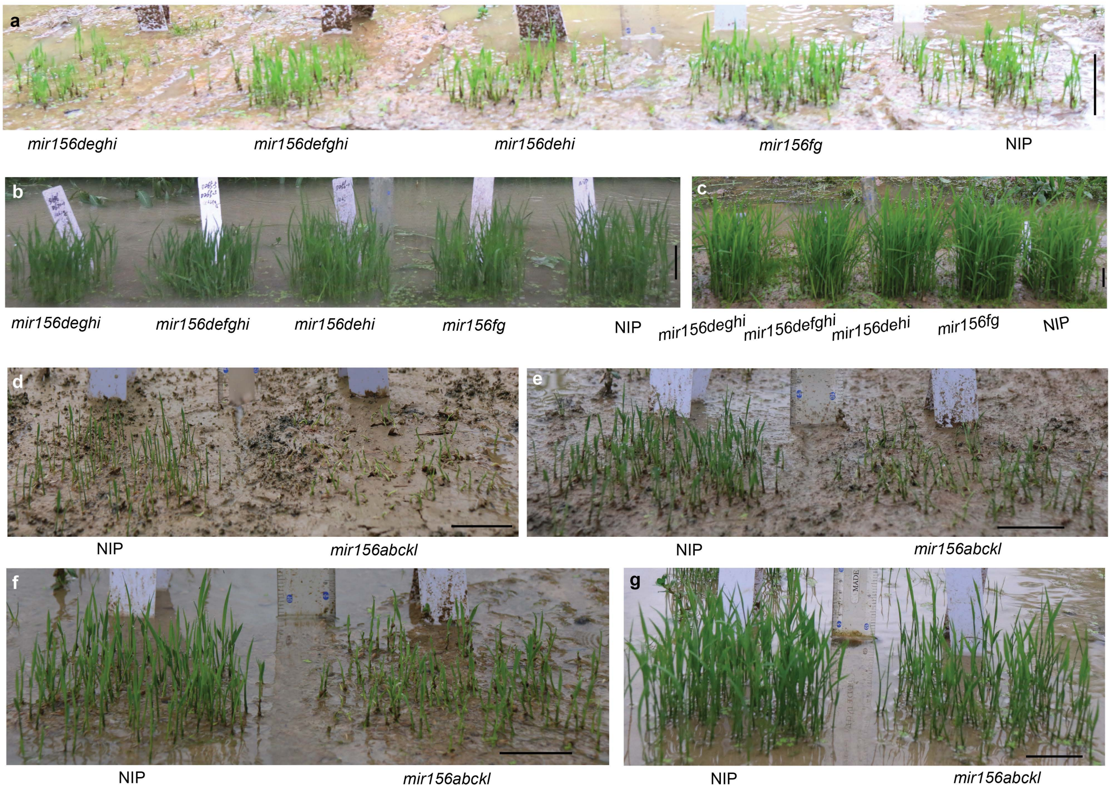

Supplementary Figure 3 Comparison of wild-type and *mir156* seedlings in Nipponbare (NIP) background. (**a**–**c**) Wild-type, *mir156fg*, *mir156dehi*, *mir156deghi* and *mir156defghi* seedlings at the sixth (**a**), tenth (**b**) and fifteenth (**c**) day after sowing. (**d**–**g**) Wild-type and *mir156abckl* seedlings at the third (**d**), fourth (**e**), fifth (**f**) and seventh (**g**) day after sowing. Scale bars, 5 cm. Before sowing, the seeds of the wild type and *mir156* mutants were soaked in water for one day, and then germinated for another day at room temperature during late June of the year 2018.

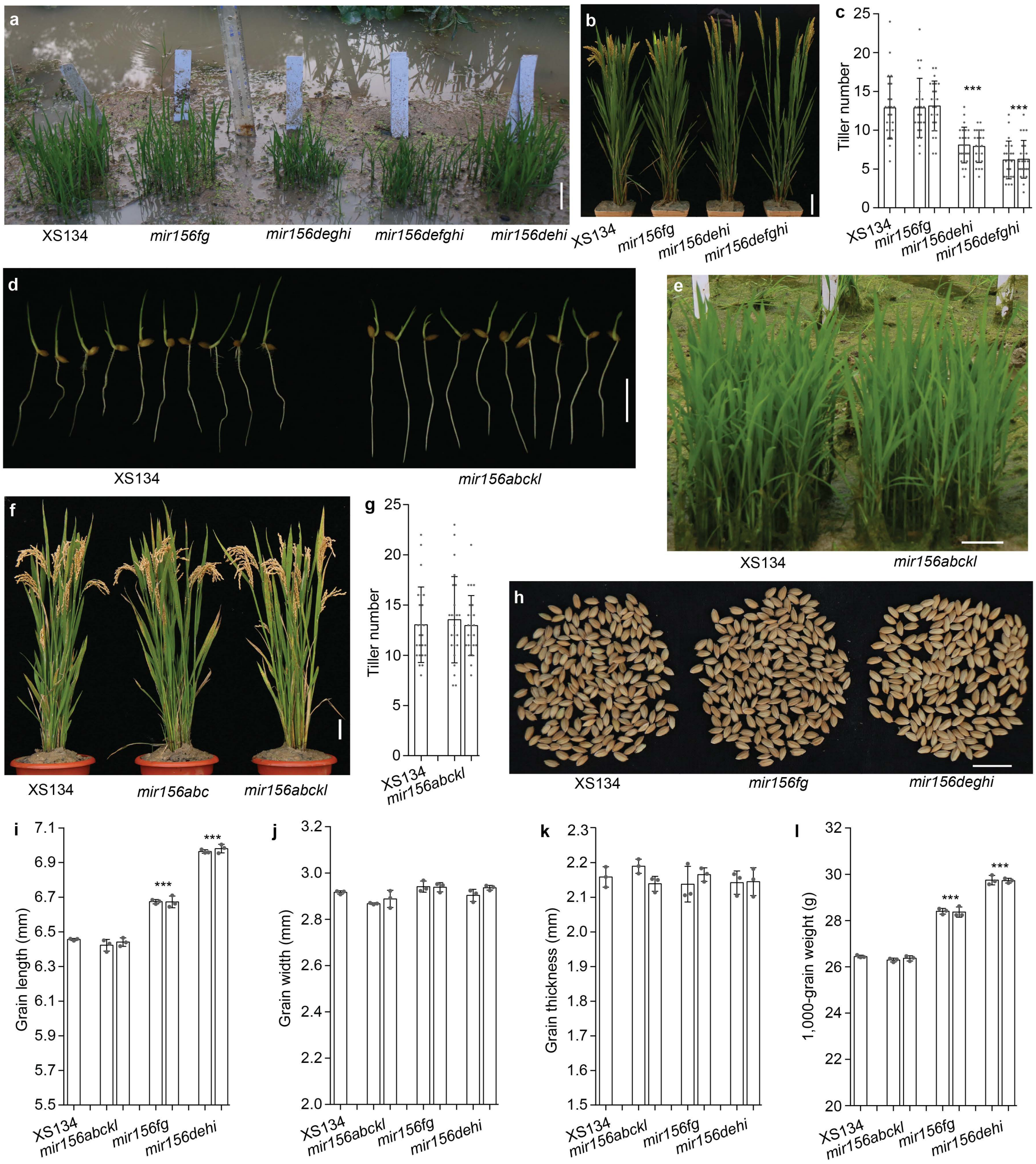

Supplementary Figure 4 Plant architectures and grain shapes of the *mir156* mutants. (**a**) Ten-day-old seedlings of the wild type, *mir156fg*, *mir156dehi*, *mir156deghi* and *mir156defghi*. Scale bar, 5 cm. (**b**) Wild-type, *mir156fg*, *mir156dehi* and *mir156defghi* plants at the mature stage. Scale bar, 10 cm. (**c**) Tiller numbers per plant of the wild type, *mir156fg*, *mir156dehi*, and *mir156defghi* at the seed-filling stage. (**d**) Wild-type and *mir156abckl* seedlings at the fifth day after germination. Scale bar, 2 cm. (**e**) 22-day-old seedlings of the wild type and *mir156abckl*. Scale bar, 5 cm. (**f**) Wild-type, *mir156abc* and *mir156abckl* plants at the mature stage. Scale bar, 10 cm. (**g**) Tiller numbers per plant of the wild type and *mir156abckl* at the seed-filling stage. (**h**) Grains of the wild type, *mir156fg* and *mir156deghi*. Scale bar, 2 cm. (**i**–**k**) Grain lengths (**i**), widths (**j**) and thicknesses (**k**) of the wild type, *mir156abckl*, *mir156fg* and *mir156dehi*. (**l**) 1000-grain weights of the wild type, *mir156abckl*, *mir156fg* and *mir156dehi*. Data are presented as means ± SD. Each bar in the bar charts represents an independent line. *P* values (versus the wild type) were calculated with Student's

t-test. ***, $P < 0.001$. Source data are provided as a Source Data file.

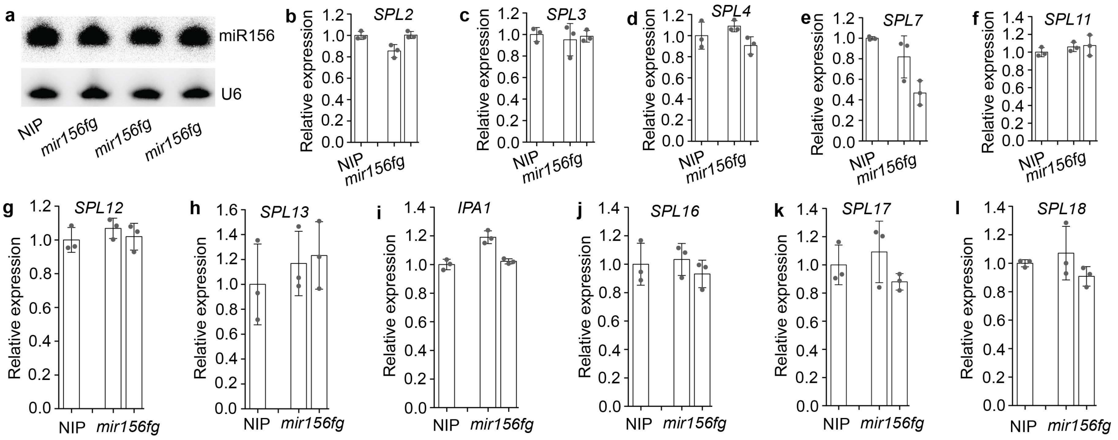

Supplementary Figure 5 Expression analyses of miR156 and its target genes in wild-type and *mir156fg* seedling shoots. (**a**) Northern blot showing miR156 abundance in wild-type and *mir156fg* seedling shoots. Three independent *mir156fg* lines were used in the Northern blotting assays. U6 RNA and miR159 were used as loading controls. (**b**–**l**) Relative expression levels of miR156 target genes in wild-type and *mir156fg* seedling shoots. Two independent *mir156fg* lines were used for the *SPL* expression analyses. Fourteen-day-old seedlings were used in the Northern blotting and *SPL* expression analyses. Three independent biological replicates were performed, and error bars indicate standard deviation. NIP, Nipponbare. Source data are provided as a Source Data file.

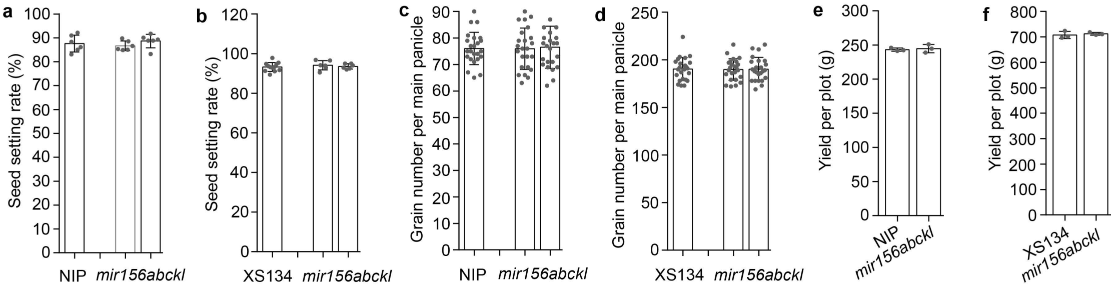

Supplementary Figure 6 Grain productivities of the wild type and *mir156abckl*. (**a** and **b**) Seed setting rates of the wild type and *mir156abckl* in Nipponbare (**a**) and XS134 (**b**) background. Six plants of the wild type and *mir156abckl* were investigated, respectively, for fertility comparison. (**c** and **d**) Grain numbers per main panicle of the wild type and *mir156abckl* in Nipponbare (**c**) and XS134 (**d**) background. (**e** and **f**) Yields per plot (90 cm x 60 cm) in Nipponbare (**e**) and XS134 (**f**) background. Data are presented as means ± SD. Each bar in the bar charts represents an independent line. NIP, Nipponbare. Source data are provided as a Source Data file.

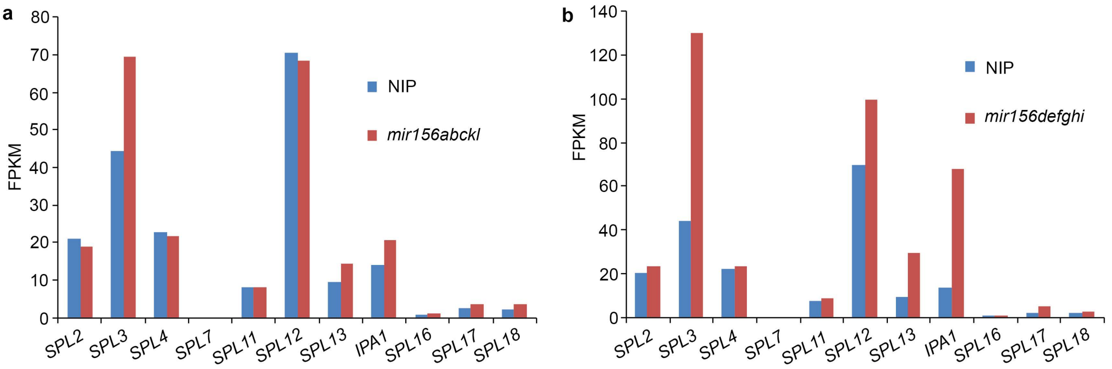

Supplementary Figure 7 Relative expression analyses of miR156 target genes in unelongated culms of 20-day-old seedlings. FPKM, fragments per kilobase of exon per million reads mapped. The data were obtained from the transcriptome analyses. The FPKM values were used to compare relative level of gene expression. Source data are provided as a Source Data file.

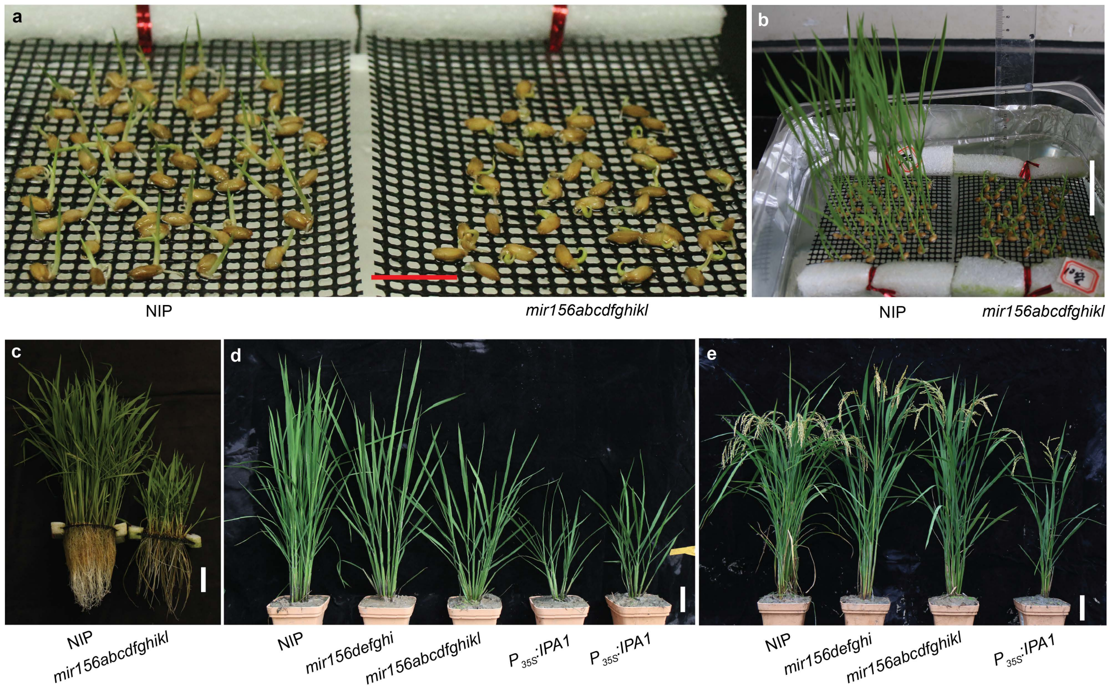

Supplementary Figure 8 Comparison of wild-type, *mir156abcdfghikl*, *mir156defghi* and *IPA1* over-expression (*P_{35S}:IPA1*) plants. (**a**–**c**) Wild-type and *mir156abcdfghikl* seedlings at the third (**a**), eighth (**b**) and twenty-third (**c**) day after germination. Scale bar in **a**, 2 cm; Scale bars in **b** and **c**, 5 cm. (**d**) 65-day-old plants of the wild type, *mir156defghi*, *mir156abcdfghikl* and two *P_{35S}:IPA1* lines. Scale bar, 10 cm. (**e**) Plants of the wild type, *mir156defghi*, *mir156abcdfghikl* and *P_{35S}:IPA1* at the mature stage. Scale bar, 10 cm. NIP, Nipponbare.

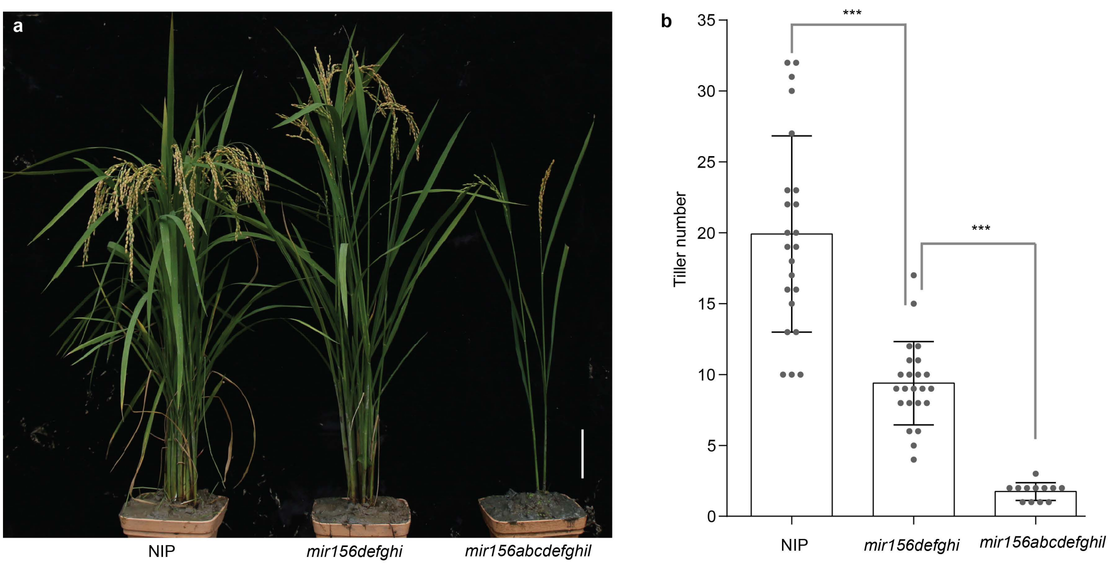

Supplementary Figure 9 Comparison of the wild type, *mir156defghi* and *mir156abcdefghil* at the mature stage. (**a**) Wild-type, *mir156defghi* and *mir156abcdefghil* plants at the mature stage. Scale bar, 10 cm. (**b**) Tiller numbers per plant of the wild type, *mir156defghi* and *mir156abcdefghil*. Data are presented as means ± SD. *P* values were calculated with Student's *t*-test. ***, $P < 0.001$. NIP, Nipponbare. Source data are provided as a Source Data file.

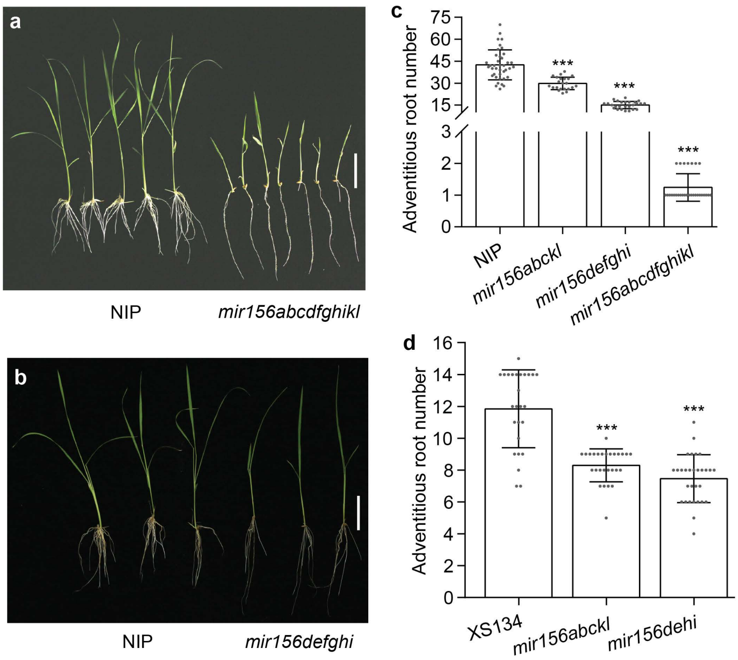

Supplementary Figure 10 *mir156* mutations suppressed adventitious root formation. (**a**) Comparison of the roots of 20-day-old wild-type and *mir156abcdfghikl* seedlings. (**b**) Comparison of the roots of two-week-old wild-type and *mir156defghi* seedlings. (**c**) Adventitious root numbers of 20-day-old wild-type, *mir156abckl*, *mir156defghi* and *mir156abcdfghikl* seedlings in Nipponbare background. (**d**) Adventitious root numbers of 10-day-old wild-type, *mir156abckl* and *mir156dehi* seedlings in XS134 background. Scale bars, 5 cm. Data are presented as means ± SD. *P* values (versus the wild type) were calculated with Student's *t*-test. ***, $P < 0.001$. NIP, Nipponbare. Source data are provided as a Source Data file.

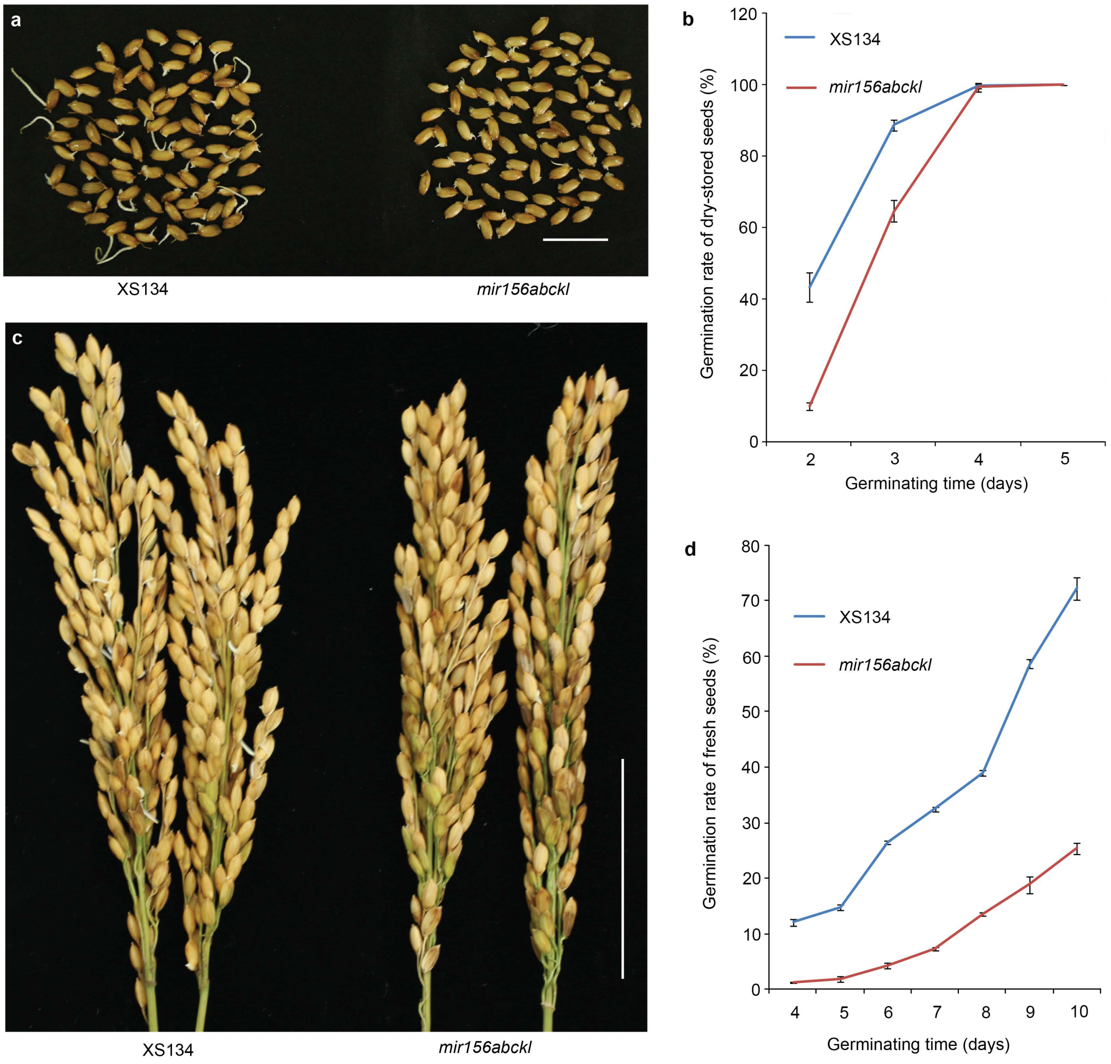

Supplementary Figure 11 *mir156abckl* mutations enhanced seed dormancy. (**a** and **b**) Germination assay of wild-type and *mir156abckl* seeds. After harvest, the seeds for this assay were immediately dried in a 42 °C dry oven for seven days, and then stored in a 20 °C dry cabinet for two weeks. Scale bar, 2 cm. (**c** and **d**) Germination assay of wild-type and *mir156abckl* fresh seeds. Scale bar, 5 cm. Data are presented as means ± SD. Source data are provided as a Source Data file.

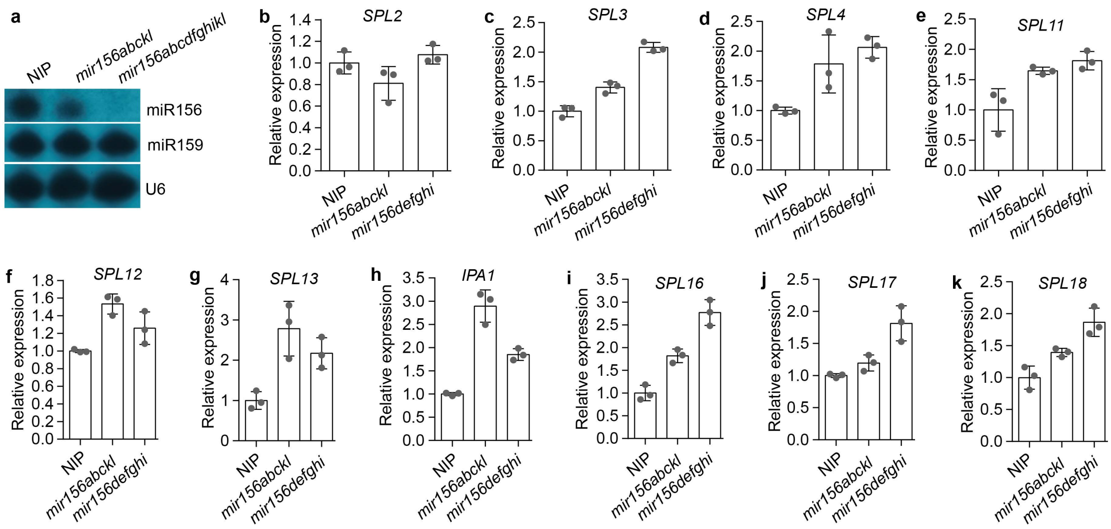

Supplementary Figure 12 Expression analyses of miR156 and its target genes in the seed embryos of *mir156* mutants. (**a**) Northern blot showing miR156 abundance in germinating seed embryos of the wild type, *mir156abckl* and *mir156abcdfghikl*. U6 RNA and miR159 were used as loading controls. Dried seeds were put in water of 30 ^{o}C for 36 hours, and then the embryos of these germinating seeds were used in the Northern blotting assays. (**b**–**k**) Relative expression levels of miR156 target genes in wild-type, *mir156abckl* and *mir156defghi* fresh seed embryos. The expression of *SPL7* was not detected in the fresh seed embryos. Three independent biological replicates were performed, and error bars indicate standard deviation. NIP, Nipponbare. Source data are provided as a Source Data file.

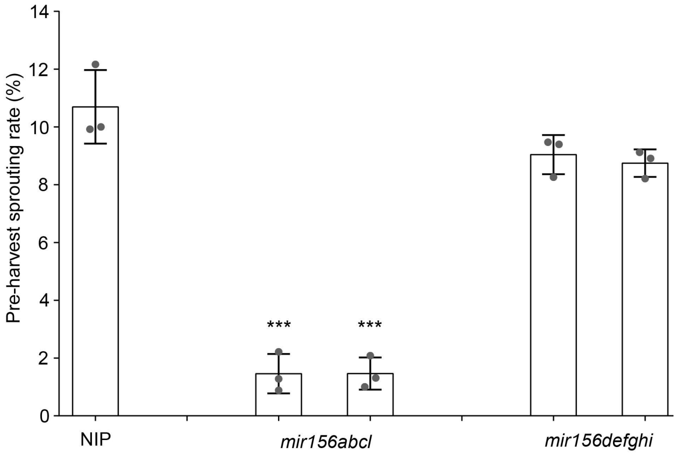

Supplementary Figure 13 PHS rates of wild-type, *mir156defghi* and *mir156abcl* seeds in Hangzhou of the year 2017. Two independent lines of every mutant type were investigated for the PHS data. Data are presented as means ± SD. *P* values (versus the wild type) were calculated with Student's *t*-test. ***, $P < 0.001$. NIP, Nipponbare. Source data are provided as a Source Data file.

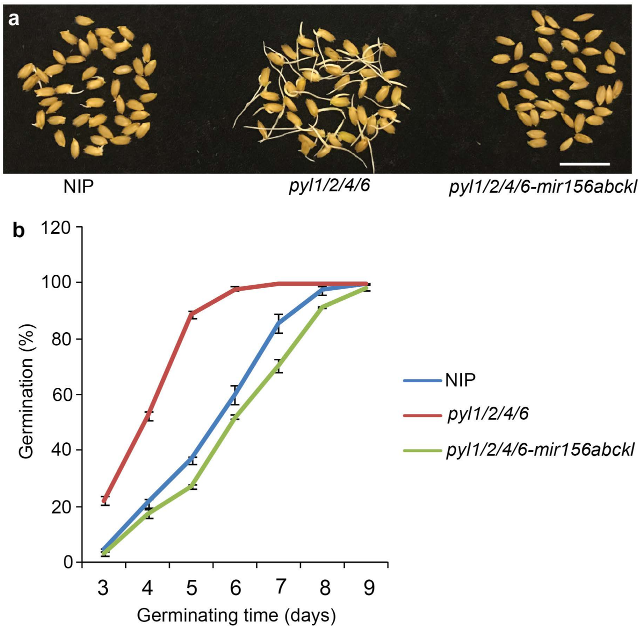

Supplementary Figure 14 *mir156abckl* mutations suppressed the seed dormancy defects caused by *pyl1/2/4/6* mutations. (**a**) Germination comparison of wild-type, *pyl1/2/4/6* and *pyl1/2/4/6-mir156abckl* fresh seeds. Scale bar, 2 cm. (**b**) Germination rates of wild-type, *pyl1/2/4/6* and *pyl1/2/4/6-mir156abckl* fresh seeds. Data are presented as means ± SD. NIP, Nipponbare. Source data are provided as a Source Data file.

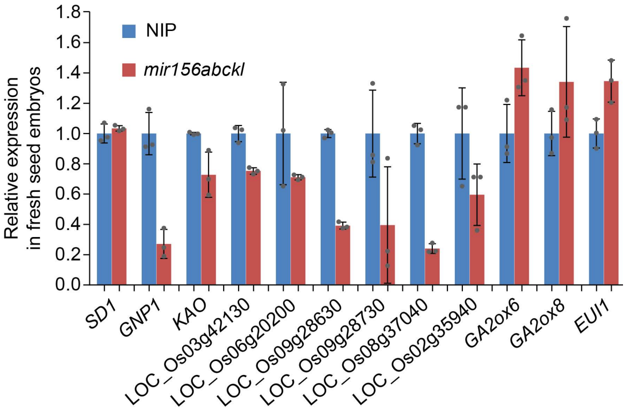

Supplementary Figure 15 Relative expression levels of several GA biosynthetic, signaling and deactivating genes in fresh seed embryos of the wild type and *mir156abckl*. Three independent biological replicates were performed, and error bars indicate standard deviation. NIP, Nipponbare. Source data are provided as a Source Data file.

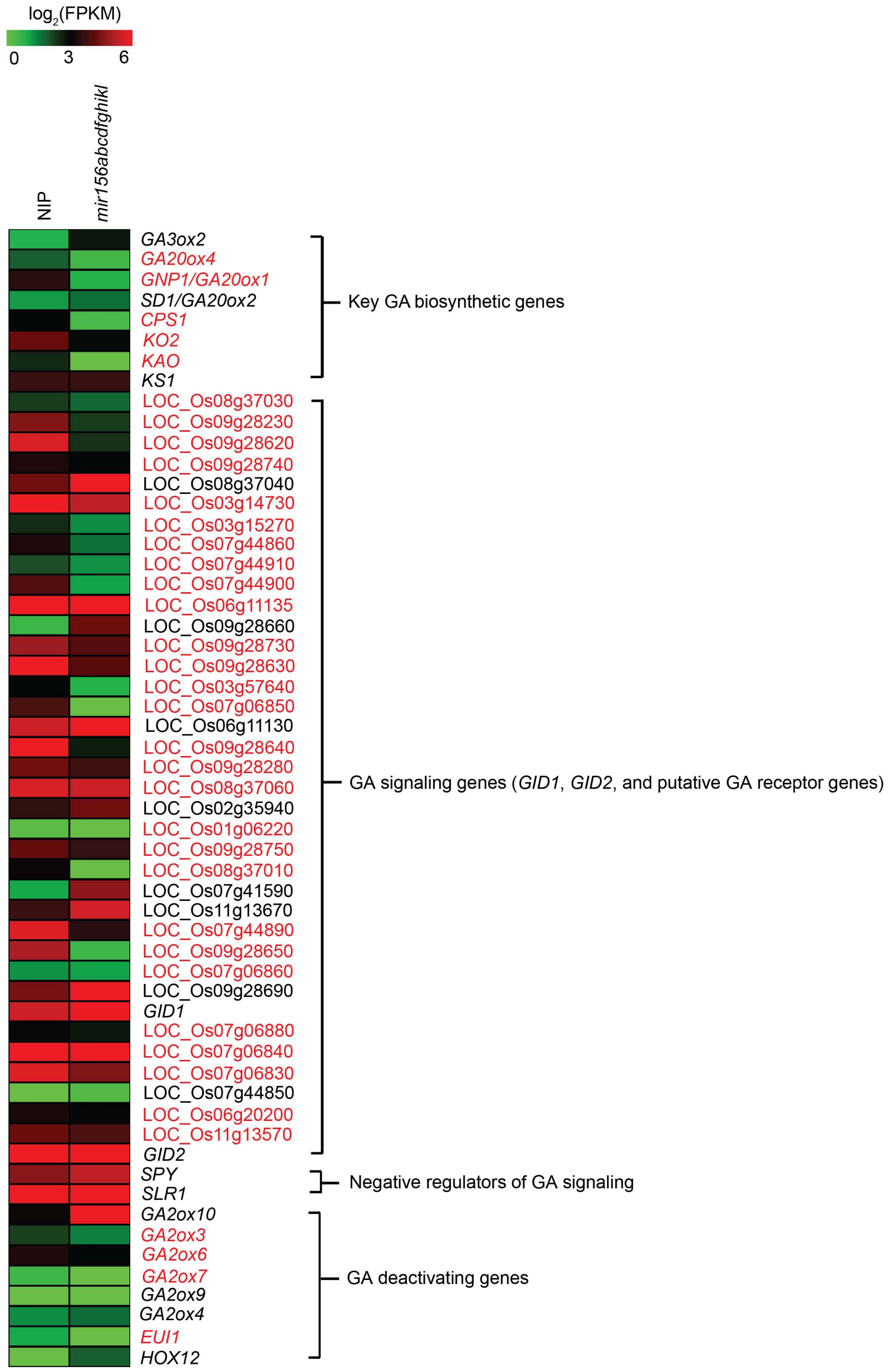

Supplementary Figure 16 Expression profiles of GA biosynthetic, signaling and deactivating genes in wild-type and *mir156abcdfghikl* seedling shoots. FPKM, fragments per kilobase of exon per million reads mapped. The genes with decreased FPKMs in *mir156abcdfghikl* are highlighted in red. The data were taken from the transcriptome analyses. NIP, Nipponbare. Source data are provided as a Source Data file.

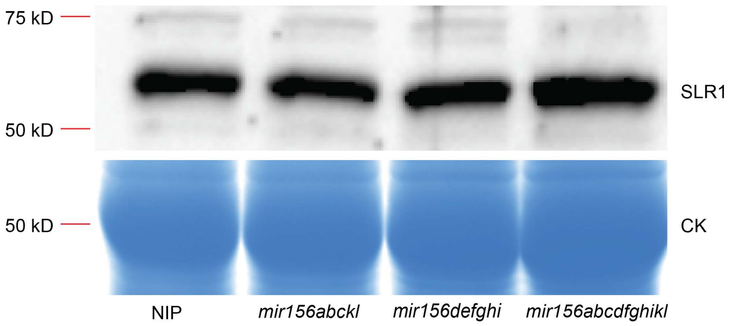

Supplementary Figure 17 Western blotting assay showing relative SLR1 protein accumulation in wild-type, *mir156abckl*, *mir156defghi*, and *mir156abcdfghikl* seedling shoots. Seven-day-old seedlings were used in the Western blotting assay. CK, the Robisco protein was used as loading control. NIP, Nipponbare.

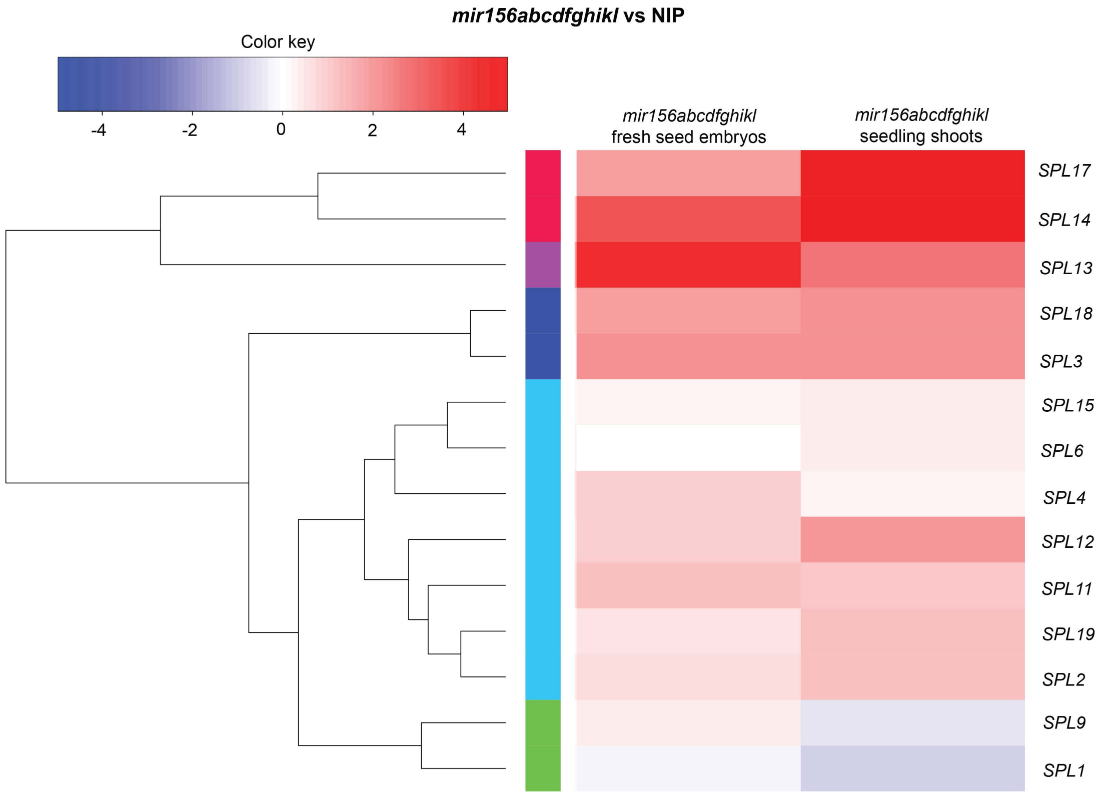

Supplementary Figure 18 The expression changes of miR156 target genes between the wild type and *mir156abcdfghikl* in fresh seed embryos and seedling shoots. NIP, Nipponbare. Color key, $\log_2$(*mir156abcdfghikl*_FPKM/NIP_FPKM); FPKM, fragments per kilobase of exon per million reads mapped. The data were taken from the transcriptome analyses. Source data are provided as a Source Data file.

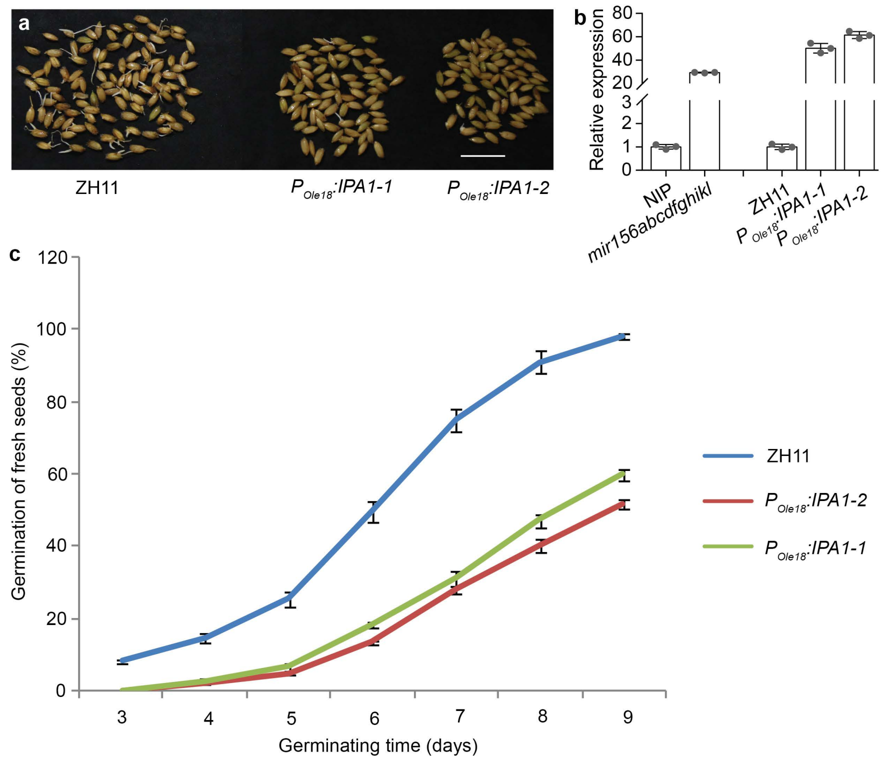

Supplementary Figure 19 Seed-specific over-expression of *IPA1* enhanced seed dormancy. (**a**) Fresh seed germinations of the wild type and two seed-specific *IPA1* over-expression (*P_{Ole18}:IPA1*) lines. Scale bar, 2 cm. (**b**) Relative expression level of *IPA1* in the fresh seed embryos of the wild type, *mir156abcdfghikl* and two *P_{Ole18}:IPA1* lines. (**c**) Germination rates of fresh seeds of the wild type and two *P_{Ole18}:IPA1* lines. Three independent biological replicates were performed, and error bars indicate standard deviation. The seed-specific over-expression lines were constructed in ZH11 background. ZH11, the *japonica* rice variety Zhonghua 11. Source data are provided as a Source Data file.

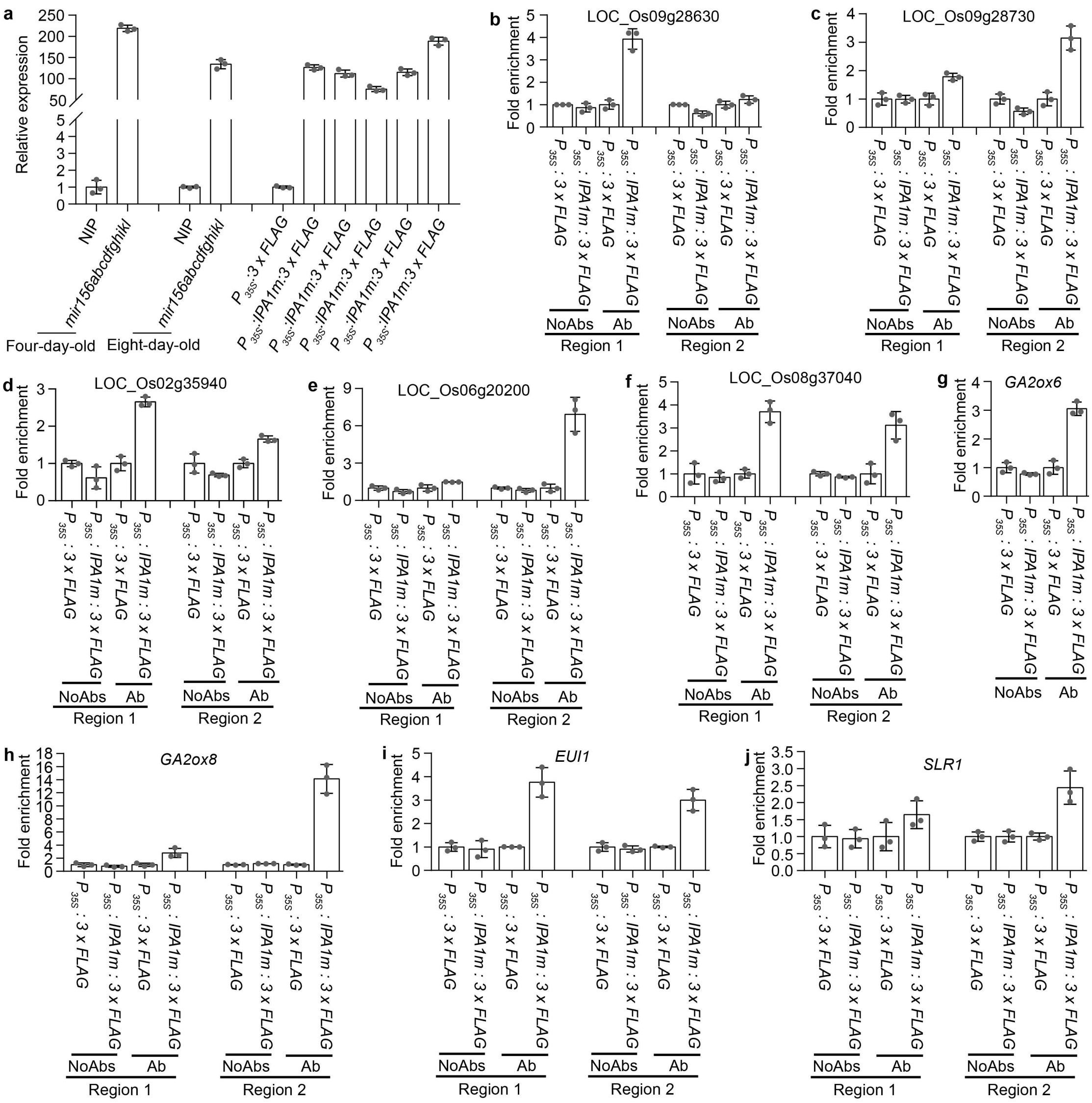

Supplementary Figure 20 ChIP-qPCR assays showing *in vivo* interactions between IPA1 and promoters of GA signaling and deactivating genes. (**a**) RT-qPCR analyses of *IPA1* expression in the seedling shoots of Nipponbare (NIP), *mir156abcdfghikl*, P_{35S}*:3×FLAG*, and P_{35S}*:IPA1m:3×FLAG* plants. Five P_{35S}*:IPA1m:3×FLAG* plants of T0 generation (about 20-day-old) were used in the expression analyses and subsequently ChIP-qPCR assays. (**b–j**) *In vivo* interactions revealed by ChIP-qPCR assays. Five 20-day-old P_{35S}*:IPA1m:3×FLAG* seedlings of T0 generation were used in these assays, and the seedling shoots of these five P_{35S}*:IPA1m:3×FLAG* plants were pooled together to conduct the ChIP-qPCR assays. IPA1m, IPA1-coding sequence with synonymous mutations at miR156-targeting site; NoAbs, without antibodies; Ab, antibodies against FLAG; regions, the fragments in the promoters detected for interactions with IPA1. Values are means ± SE (n = 3). The fold enrichment was normalized against the promoter of *UBIQUITIN*. Source data are provided as a Source Data file.

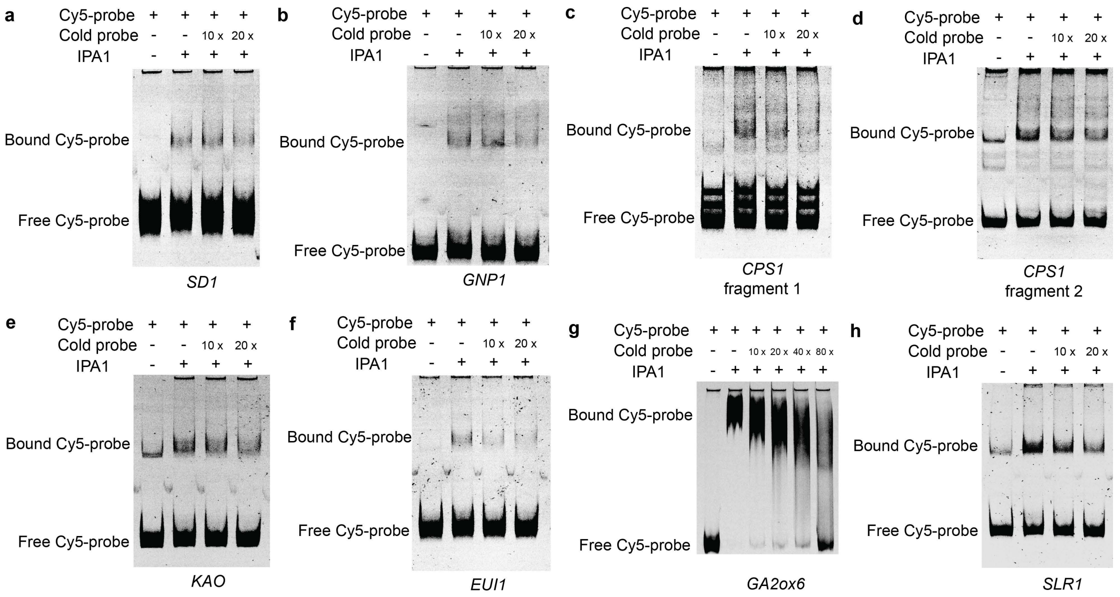

Supplementary Figure 21 Direct binding of IPA1 to the promoters of seven GA-related genes. The 10-, 20-, 40-, or 80-fold excess nonlabeled probes were used for competition assays. Fragments, the promoter fragments tested for interactions with IPA1; Cy5-probe, Cy5-labeled probe; cold probe, unlabeled probe. Source data are provided as a Source Data file.

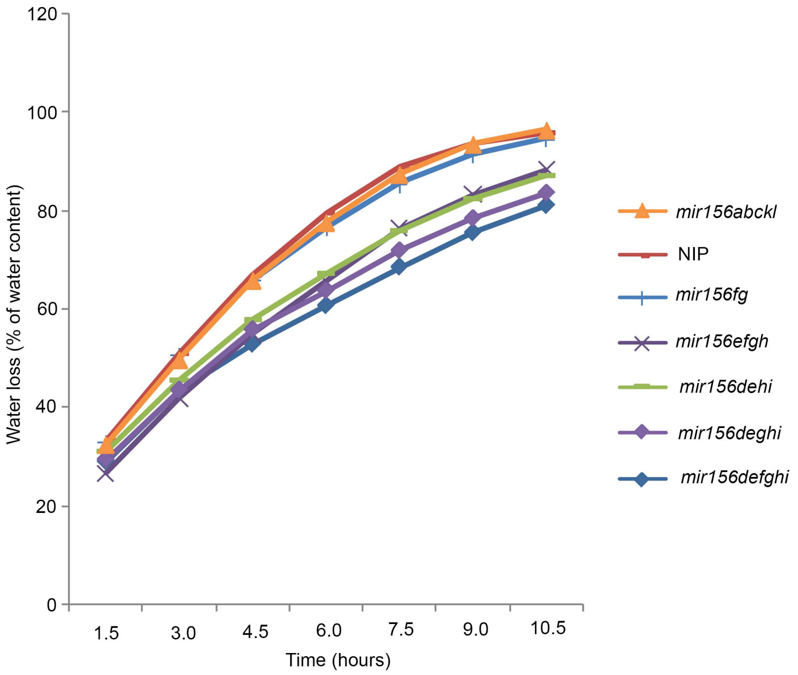

Supplementary Figure 22 Cumulative transpirational water loss from the detached flag leaf blades of the wild type, *mir156abckl*, *mir156fg*, *mir156efgh*, *mir156dehi*, *mir156deghi*, and *mir156defghi*. For water loss assays, detached flag leaf blades were placed in room temperature and weighed at indicated time points. NIP, Nipponbare. Source data are provided as a Source Data file.

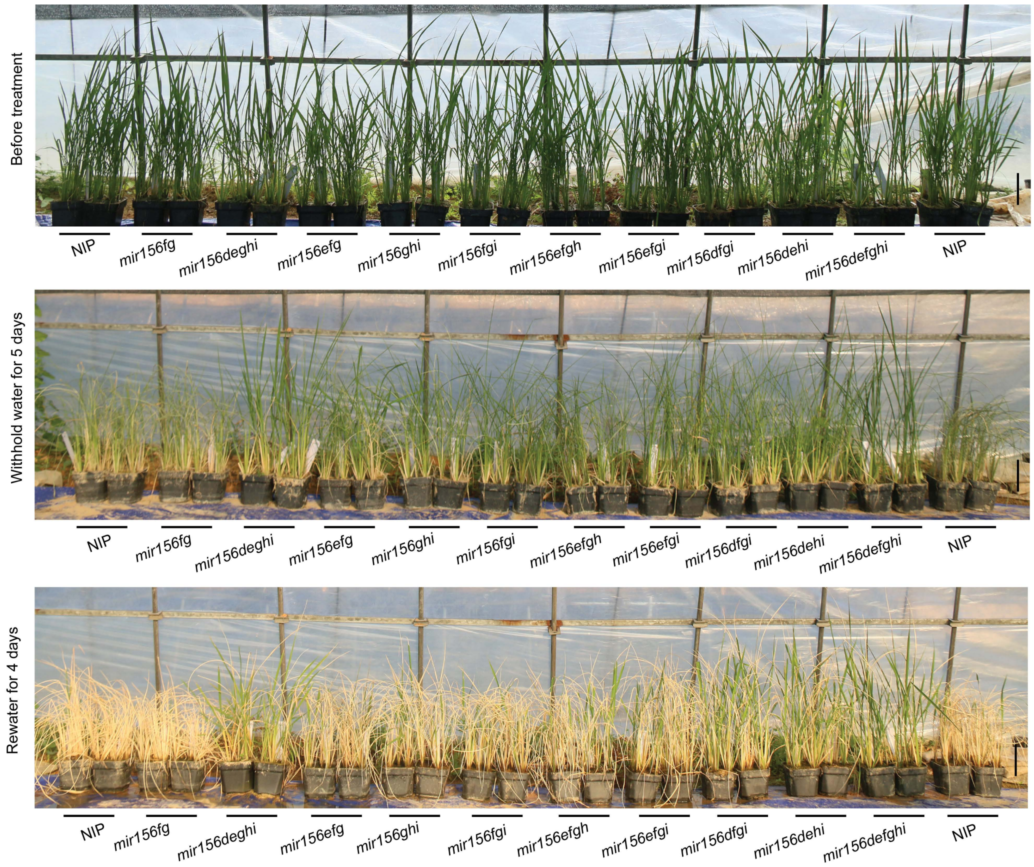

Supplementary Figure 23 Drought stress assays of wild-type, *mir156fg*, *mir156efg*, *mir156ghi*, *mir156fgi*, *mir156efgh*, *mir156efgi*, *mir156dfgi*, *mir156dehi*, *mir156deghi*, and *mir156defghi* plants. Thirty-day-old plants were used for the drought stress assays. NIP, Nipponbare. Scale bars, 10 cm.

Supplementary Table 1 Tiller numbers, plant heights, and spikelet numbers per main panicle of the wild type and group I *mir156* mutants

Genotype	No. of plants	Tiller number per plant	Plant height (cm)	No. of spikelets
mir156defghi	20	9.3 ± 2.3[c]	88.97 ± 2.85[c]	60.9 ± 5.9[a]
mir156defghi	22	9.0 ± 1.7[c]	88.29 ± 1.88[c]	67.6 ± 12.1
mir156defgh	20	10.2 ± 3.4[c]	88.88 ± 2.97[c]	63.1 ± 10.5
NIP	23	21.4 ± 4.9	77.86 ± 2.83	67.7 ± 9.4
mir156defgh	21	8.9 ±2.9[c]	86.62 ± 2.57[c]	60 ± 4.6[b]
mir156defgi	21	13 ± 3.8[c]	84.71 ± 2.25[c]	67 ± 10.7
mir156defgi	20	11 ± 5.2[c]	85.62 ± 3.99[c]	68.1 ± 8.8
mir156dehi	24	11.9 ± 6.3[c]	88.67 ± 1.85[c]	70.2 ± 8.4
mir156dehi	19	12.6 ± 3.9[c]	88.99 ± 1.42[c]	70.7 ± 6
mir156dfgi	20	14.9 ± 6.3[c]	85.64 ± 2.46[c]	72.1 ± 5.3
NIP	20	22 ± 5.3	77.81 ± 3.21	71.8 ± 9.8
mir156dfgi	20	12.9 ± 5.9[c]	85.44 ± 2.25[c]	68.7 ± 7.2
mir156efgi	21	13.1 ± 3[c]	84.62 ± 3.76[c]	72.7 ± 11.2
mir156efgi	23	10.3 ± 2.7[c]	85.17 ± 3.71[c]	81.7 ± 9.2[b]
mir156efgh	18	15.3 ± 4.3[c]	87.18 ± 3.13[c]	74 ± 8.4[b]
mir156efgh	18	11.78 ± 3.7[c]	85.82 ± 3.48[c]	70.6 ± 8.5
mir156eghi	21	13.86 ± 3.8[c]	77.76 ± 1.9[b]	64.56 ± 8.4
NIP	18	21.89 ± 4.8	75.07 ± 3.2	65.4 ± 10.5
mir156dghi	21	14 ± 5.7[c]	87.29 ± 2.82[c]	78.1 ± 12[b]
mir156ghi	23	13.48 ± 5.1[c]	83.9 ± 2.5[c]	71 ± 11
mir156efg	19	18.26 ± 5.1[a]	76.28 ± 3.03	65.7 ± 10.2
mir156efg	19	16.74 ± 7.6[a]	76.43 ± 2.63	61.1 ± 8.4
mir156fgi	18	19.5 ± 6.1	74.17 ± 3.99	63.5 ± 6.9[a]
mir156ei	24	17.13 ± 4.8[b]	73.01 ± 2.85	58.1 ± 8.3[c]
NIP	18	21 ± 4.6	76.84 ± 2.85	69.3 ± 7.8
mir156fg	19	19.1 ± 5	77.72 ± 2.69	69.2 ± 6
mir156fg	20	20.2 ± 7	76.89 ± 2.31	73 ± 10.2

All data are present as means ± SD; *t*-test was used to compare the values between the wild type and *mir156* mutants; [a, b, c] statistically significant at $P < 0.05$, 0.01 and 0.001 respectively; 3 largest panicles per plant were investigated for the No. of spikelets per main panicle ($18 \leq n \leq 24$); NIP, Nipponbare. Source data are provided as a Source Data file.

Supplementary Table 2 Expression profiles of miR156 target genes in unelongated culms of wild-type and *mir156abckl* seedlings

Genes	NIP_FPKM	*mir156abckl*_FPKM	Log_2(*mir156abckl* / NIP)	*P*_value	*q*_value
SPL2	21.0883	19.2491	-0.131655	0.0996	0.166383
SPL3	44.47	69.722	0.648781	0.00005	0.000182374
SPL4	22.8996	21.8477	-0.0678436	0.51415	0.620702
SPL7	—	—	—	—	—
SPL11	8.18178	8.35533	0.0302814	0.7735	0.836478
SPL12	70.4663	68.4109	-0.0427064	0.52385	0.629447
SPL13	9.83188	14.6551	0.575866	0.00005	0.000182374
IPA1	14.1521	20.8426	0.558513	0.00005	0.000182374
SPL16	0.954843	1.32208	0.46947	0.0386	0.0745171
SPL17	2.62183	3.7407	0.512732	0.00125	0.00357331
SPL18	2.54522	3.67932	0.531651	0.0017	0.00471343

The data were taken from the transcriptome analyses. —, undetectable expression; NIP, Nipponbare. Source data are provided as a Source Data file.

Supplementary Table 3 Expression profiles of miR156 target genes in unelongated culms of wild-type and *mir156defghi* seedlings

Genes	NIP_FPKM	*mir156defghi*_FPKM	Log_2(*mir156defghi* / NIP)	*P*_value	*q*_value
SPL2	20.9733	23.5354	0.16628	0.0849	0.157669
SPL3	44.2086	130.01	1.55622	0.00005	0.00022456
SPL4	22.7726	23.4414	0.0417546	0.7177	0.802728
SPL7	—	—	—	—	—
SPL11	8.13516	8.8555	0.122404	0.3123	0.437697
SPL12	70.0645	99.6751	0.508549	0.00005	0.00022456
SPL13	9.78071	29.9168	1.61295	0.00005	0.00022456
IPA1	14.0763	68.2081	2.27668	0.00005	0.00022456
SPL16	0.949682	1.05124	0.14657	0.5526	0.665897
SPL17	2.60693	5.23315	1.00533	0.00005	0.00022456
SPL18	2.53177	3.00986	0.249552	0.17115	0.274987

The data were taken from the transcriptome analyses. —, undetectable expression; NIP, Nipponbare. Source data are provided as a Source Data file.

Supplementary Table 4 Expression profiles of miR156 target genes in wild-type and *mir156abcdfghikl* fresh seed embryos

Genes	NIP_FPKM	*mir156abcdfghikl*_FPKM	Log_2(*mir156abcdfghikl* / NIP)	*P*_value	*q*_value
SPL2	0.542494	0.836622	0.624968	0.13565	0.478693
SPL3	5.5307	23.1781	2.06723	0.00005	0.00139824
SPL4	4.44413	8.29227	0.899867	0.00015	0.00357622
SPL7	—	—	—	—	—
SPL11	2.52843	5.54622	1.13326	0.0001	0.00255101
SPL12	8.42813	15.6035	0.888588	0.00005	0.00139824
SPL13	0.123262	2.37699	4.26934	0.00055	0.0100066
IPA1	8.6931	86.8622	3.32079	0.00005	0.00139824
SPL16	—	—	—	—	—
SPL17	2.453	9.17608	1.90333	0.00005	0.00139824
SPL18	0.947711	3.54328	1.90257	0.00005	0.00139824

The data were taken from the transcriptome analyses. —, undetectable expression; NIP, Nipponbare. Source data are provided as a Source Data file.

Supplementary Table 5 Expression profiles of miR156 target genes in wild-type and *mir156abcdfghikl* seedling shoots

Genes	NIP_FPKM	*mir156abcdfghikl*_FPKM	Log_2(*mir156abcdfghikl* / NIP)	*P*_value	*q*_value
SPL2	12.397	28.3187	1.19176	0.00005	0.0000993302
SPL3	18.4319	82.4889	2.16199	0.00005	0.0000993302
SPL4	12.2986	14.499	0.237451	0.17765	0.214112
SPL7	0.0812527	1.16495	3.84171	0.0032	0.0051636
SPL11	5.60632	11.7975	1.07336	0.00005	0.0000993302
SPL12	16.7684	64.1154	1.93493	0.00005	0.0000993302
SPL13	5.10738	33.5657	2.71633	0.00005	0.0000993302
IPA1	1.07335	33.7871	4.97628	0.00005	0.0000993302
SPL16	0.265821	3.24186	3.60829	0.00005	0.0000993302
SPL17	0.378972	10.0147	4.72388	0.00005	0.0000993302
SPL18	2.45144	11.146	2.18483	0.00005	0.0000993302

The data were taken from the transcriptome analyses. NIP, Nipponbare. Source data are provided as a Source Data file.

Supplementary Table 6 Probes used in the Northern blotting assays

Probes	Sequences (5’ to 3’)
anti-miR156	GTGCTCACTCTCTTCTGTCA
anti-miR159	TGGAGCTCCCTTCAATCCAAT
U6	TGTATCGTTCCAATTTTATCGGATGT

Supplementary Table 7 Primers for RT-qPCR

Genes	Primers	Sequences (5' to 3')
GNP1	QGNP1-1F	ATCATGCGCCTCAACTACTAC
	QGNP1-1R	TTGGAGAGCGCCATGAAGGTG
SD1	QSD1-F	CAATGGGGAGGGTGTACCAGA
	QSD1-R	TCCTGGAGGAGGATGGTGAG
GA20ox4	QGA20ox4-F	CTAGGCGACGACTTCAAACAC
	QGA20ox4-R	GTAGTAGTTGCACCGCATGATC
KAO	QKAO-F	TTCGCAAGAGCAAAGGCTGAG
	QKAO-R	GGAAGGACACGAAGGAGATG
CPS1	QCPS1-F	ATGTCAAGCATGAAAAGGATGG
	QCPS1-R	ACTTTTGACCTTCTCGAACTG
KO2	QKO2-F	TAGCCAAGGAGGCGATGGTTG
	QKO2-R	TGTCACGAAATTGTTTCTGTGC
LOC_Os03g42130	Q42130-F	CAGAGGACGTTCTTCGCGCT
	Q42130-R	CCATTGCCTCCCTGAATCCC
LOC_Os06g20200	Q20200-F	TGTGATACTCCTGGAGCCGTA
	Q20200-R	GGAACGCTTCTTGCAGCTCG
LOC_Os09g28630	Q28630-F	ATGGCCTCCGAGACCGAGC
	Q28630-R	CCTCCGTGGAAGAAGACGAG
LOC_Os09g28730	Q28730-F	CTCCTCCACCCCTACTTCTG
	Q28730-R	AGCAGTGCTCCGCCGTAGTG
LOC_Os08g37040	Q37040-F	CACTGCTACTTCCTCAACAACC
	Q37040-R	ATGGCTTTACTCGGGTGTGTAG
LOC_ Os02g35940	Q35940-F	GAGATCCCAAGAAGACACGAG
	Q35940-R	CTCTTGTAGACCCGGAGGATC
SLR1	QSLR1-F	TTGCAGCAGGTGGGTTGGAAG
	QSLR1-R	AGTTGACGGCGATCACCTCAG
SPY	QSPY-F	CGAGTTGACTTGCTGCCACTCAT
	QSPY-R	CCATAGTAACACATGGAACCCCCA
GA2ox6	QGA2ox6-F	CACCTCCTTAAGGGGCGTGA
	QGA2ox6-R	GACGGTGAGGAAGTCGCTGT
GA2ox8	QGA2ox8-F	CCCGATTCCTTCTTCGTCAAC
	QGA2ox8-R	TCCCTGTACAGGCTCTGCTC
GA2ox10	QGA2ox10-F	AGGAGAAAGATGCGTTGGTTAGG
	QGA2ox10-R	CGGAGCACCGAGATGATCTGA
EUI1	QEUI1-F	AAGCCTACGGCGAGACGTAC
	QEUI1-R	CACGGGCCATGTAGAACTCC
SPL2	SPL2-F	TGCGAGGCGCACTCCAAGA
	SPL2-R	CGAGGTGAATCTTGCCGCT
SPL3	SPL3-F	CAGTGTAGTCGGTTTCACGG
	SPL3-R	CTTGCACAGCTTCCCATCTG
SPL4	SPL4-F	AGTGCAGCCGGTTCCATGC
	SPL4-R	CTTACATGGCTGAGTGGAGC

SPL7	SPL7-F	ATGCAGCCGGTTCCACGTG
	SPL7-R	TGGCTCTAGCCGATGACAGA
SPL11	SPL11-F	GCAGTGCAGCAGGTTCCAC
	SPL11-R	GTCAGATGTCCACGAGGAAC
SPL12	SPL12-F	CAGTGTAGTCGGTTTCATGGT
	SPL12-R	TCCCAGGTTGTCACATGAAGA
SPL13	SPL13-F	CTGCTGGCGCGTCTTCTAG
	SPL13-R	GCTCATGGAACCGGCTGCA
IPA1	IPA1-F	GCATCTGTTGGTGAGCATCG
	IPA1-R	TGGCCCTGAAGAAGAGCTAC
SPL16	SPL16-F	GAGATGCGCTTCTGCCAGC
	SPL16-R	GAATCTTGCCCCTTGTTGGC
SPL17	SPL17-F	CATCCTTTCATGAAGAGCCAG
	SPL17-R	GATCACTGTATCCCGCAACTG
SPL18	SPL18-F	CTAAGCGTAGTTGTAGAAAACG
	SPL18-R	GTCTCCTCGGATTTGATGATC
UBIQUITIN	Ubi-F	AACCAGCTGAGGCCCAAGA
(LOC_Os03g13170)	Ubi-R	ACGATTGATTTAACCAGTCCATGA

Supplementary Table 8 Primers for ChIP-qPCR

Genes	Target regions	Primers	Sequences (5' to 3')
CPS1	Region 1: -358···-183	CPS1-1F	TTTTGCTGTATATACGTCGGTG
		CPS1-1R	AGCCCCATACCTATCCAAACC
	Region 2: -773···-611	CPS1-2F	CTATCGCTACACACCACATAC
		CPS1-2R	ATTTGGTCAAACTTAAATCCGC
KAO	Region 1: -537···-375	KAO-1F	GGTTAATCAGTTTTGCATTTCG
		KAO-1R	ATTATTCATCGTGAGCATGTCC
	Region 2: -980···-779	KAO-2F	GTAAAAGCTTTAGTACCAATCC
		KAO-2R	ACTTGCGTATAGACTGTACTG
KO2	-484···-294	KO2-F	GTGATTTGTTTCATGGAACCG
		KO2-R	GCTTAGGGAATATTGTTAGTAC
SD1	Region 1: -221···-56	SD1-1F	TTGCATGGGGGTCATTGATTCG
		SD1-1R	TGTGAGTGTGAGTGTGTGTGTG
	Region 2: -774···-613	SD1-2F	TCGCTTAGATTCCGCATCGTC
		SD1-2R	CTTTCTTGGGCATTTGCTACTC
GNP1	-847···-701	GNP1-F	GTGAAGTGGTTGTCTAAATCTC
		GNP1-R	GTAGTTCTGAGTTTTTCACTTGG
GA20ox4	-704···-502	GA20ox4-F	TATATTTTATTCATTTCGGTAGAACC
		GA20ox4-R	TCAGTGATTGAAATTTCGCCAC
GA2ox6	-473···-257	GA2ox6-F	TGTTTGTACAACTTAAGGTTCTG
		GA2ox6-R	GGTTATGGCTGGGCCTTTGGT
GA2ox8	Region 1: -135···-4	GA2ox8-1F	GAGCAGAACACAAGTGCAAACG
		GA2ox8-1R	GCGTGATCAGATCAAAAACCAC
	Region 2: -1318···-1154	GA2ox8-2F	AAAGGGAGAAACAAAATCATTGG
		GA2ox8-2R	CAATGTGTGACTTTGTTTGCATG
GA2ox10	-583···-395	GA2ox10-F	GTTTGTCAAAAATATTATACTATGTGAG
		GA2ox10-R	GTGTATGAGGATGACGATGATG
EUI1	Region 1: -1026···-821	EUI1-1F	CTAACCTAACCTAGTGGTACTAC
		EUI1-1R	GTCATCACGAGTCCAAAATCAC
	Region 2: -424···-257	EUI1-2F	CAGATTAAACTGCACGAATTACG
		EUI1-2R	CTGTGCAAAACCGCGAGTGG
SLR1	Region 1: -618···-366	SLR1-1F	GGTACAAAGAGATATCAGGATG
		SLR1-1R	GATCGATCGATGTACGGATTC
	Region 2: -992···-817	SLR1-2F	GTATGCATAGAGTGTGTACGTG
		SLR1-2R	GCCAATGCATCTTACAAAACTG
LOC_Os09g28630	Region 1: -608···-438	28630-1F	TATGTTACCACCGTCAATCAGC
		28630-1R	GACCACACCGGGTTTTTCTCC
	Region 2: -161···-1	28630-2F	AAGATCCAAGGACGAAAAAGG
		28630-2R	GGCGAATTGGTGGATGCTGC
LOC_Os09g28730	Region 1: -689···-530	28730-1F	TTGAATGTCGTGTAAATAGTTCC
		28730-1R	AACAGACCATGAATTTTGGAGG
	Region 2: -1328···-1141	28730-2F	TGCAAATGGGCCATCCTTATTG
		28730-2R	TTTCCTTCAGTGTAAGACTCTG

LOC_Os02g35940	Region 1: -218···-44	35940-1F	GTCTTGGTAATACTAATATGGTAC
		35940-1R	AGGAGGAGGAGAATTTGTCTG
	Region 2: -749···-562	35940-2F	TCGTCGTACGCAGCAGGGAAC
		35940-2R	GAAGAGTGAGAACGTACGGAGG
LOC_Os06g20200	Region 1: -476···-262	20200-1F	CTTCAGTCAAGATGTACCCTG
		20200-1R	ACGCTAGCTACGGCCCATGT
	Region 2: -206···-45	20200-2F	CCAAATCGATTTCACATCATTCG
		20200-2R	AAGCACTGAGTACGCTGCAC
LOC_Os08g37040	Region 1: -133···+29	37040-1F	CACTACGTGCGCCACGTACATC
		37040-1R	AGCAACGCGAACAGCGACCG
	Region 2: -630···-400	37040-2F	GGGTCTCTTAAATGACCAGTG
		37040-2R	TTGCTGCCTACGTACAACGAC
	Region 3: -987···-828	37040-3F	TTTCCAACGGCAAGATGCTAG
		37040-3R	TGCTAACCGAAAGCAGCCGAG
UBIQUITIN (LOC_Os03g13170)	-911···-821	Ubpro-F	TCGGAGACCGTGCTAGGTTT
		Ubpro-R	GCCAGCGCCCATCGATT

Supplementary Table 9 Primers used in EMSAs

Genes	Primers	Regions in the promoters	Sequences (5' to 3')
GNP1	GNP1-p-F	Fragment 2:	AGAAGGTAGCTAGGGGAGAGA
	GNP1-p-R	-290···-51	GATAGATGCAACCTACAACTAAC
SD1	SD1-p-F	Fragment:	GAGCCCAAGTGGGTACGGTT
	SD1-p-R	-1102···-680	AGCTAGTACCACAAGCTAGTC
SLR1	SLR1-p2-F	Fragment:	GGGTACAAAGAGATATCAGGAT
	SLR1-p2-R	-619···-368	TCGATCGATGTACGGATTCGT
EUI1	EUI1-p2-F	Fragment:	GCACATAGTATCTAACCTAACC
	EUI1-p2-R	-1037···-822	TCATCACGAGTCCAAAATCACA
GA2ox6	GA2ox6-p-F	Fragment 1:	CTCCTAGGAGTGTCGCACC
	GA2ox6-p-R	-233···-21	AGGCGAGAGGCTTATCTGTG
KAO	KAO-p-F	Fragment:	CACTGGAATTGATGGACTGTG
	KAO-p-R	-513···-306	TTGTTCTCGAACCCTCAACAC
CPS1	CPS1-p1-F	Fragment 1:	AGTACAATACTACATAGCACAGT
	CPS1-p1-R	-1377···-1167	GTCCCTTGTCTAACATAGTAC
	CPS1-p2-F	Fragment 2:	TGTCCACTACCCCACCTTTCT
	CPS1-p2-R	-832···-639	TTGACCAAAGTCAACCTGAAAC

Supplementary Data 1 The list of rice *mir156* mutants. **a** The list of rice *mir156* mutants in Nipponbare background. **b** The list of rice *mir156* mutants in XS134 background. -, one base pair deletion; red color indicates insertion; blue color indicates base pair replacement.

Supplementary Data 2 Gene expression profiles in wild-type and *mir156abcdfghikl* fresh seed embryos. NIP, Nipponbare; C403, *mir156abcdfghikl*.

Supplementary Data 3 Expression profiles of the DEGs identified in wild-type and *mir156abcdfghikl* fresh seed embryos. NIP, Nipponbare; C403, *mir156abcdfghikl*. Ratio ⩾ 2 or ⩽ 0.5, and FDR ＜ 0.05.

Supplementary Data 4 Expression profiles of the GA biosynthetic, signaling and deactivating DEGs identified in wild-type and *mir156abcdfghikl* fresh seed embryos. NIP, Nipponbare; C403, *mir156abcdfghikl*. Ratio ⩾ 1.5 or ⩽ 0.75.

Supplementary Data 5 Gene expression profiles in wild-type and *mir156abcdfghikl* seedling shoots. NIP, Nipponbare; D216, *mir156abcdfghikl*.

Supplementary Data 6 Expression profiles of the GA biosynthetic, signaling and deactivating genes in wild-type and *mir156abcdfghikl* seedling shoots. NIP, Nipponbare; D216, *mir156abcdfghikl*.

Supplementary Data 7 Expression profiles of the GA biosynthetic, signaling and deactivating DEGs in wild-type and *mir156abcdfghikl* seedling shoots. NIP, Nipponbare; D216, *mir156abcdfghikl*. Ratio ⩾ 2 or ⩽ 0.5.
