# Supplementary material for: The grain yield modulator miR156 regulates seed dormancy through the gibberellin pathway in rice
Source: Nat Commun. 2019 Aug 23;10:3822. doi: 10.1038/s41467-019-11830-5 (PMC6707268; doi:10.1038/s41467-019-11830-5)
Supplement: Supplementary file 3 — Description of Additional Supplementary Files [file 41467_2019_11830_MOESM3_ESM.pdf]

Description of Additional Supplementary Files

File Name: Supplementary Data 1
Description: The list of rice mir156 mutants. a The list of rice mir156 mutants in Nipponbare background. b The list of rice mir156 mutants in XS134 background. -, one base pair deletion; red color indicates insertion; blue color indicates base pair replacement.

File Name: Supplementary Data 2
Description: Gene expression profiles in wild-type and mir156abcdfghikl fresh seed embryos. NIP, Nipponbare; C403, mir156abcdfghikl.

File Name: Supplementary Data 3
Description: Expression profiles of the DEGs identified in wild-type and mir156abcdfghikl fresh seed embryos. NIP, Nipponbare; C403, mir156abcdfghikl. Ratio ≥ 2 or ≤ 0.5, and FDR $<$ 0.05.

File Name: Supplementary Data 4
Description: Expression profiles of the GA biosynthetic, signaling and deactivating DEGs identified in wild-type and mir156abcdfghikl fresh seed embryos. NIP, Nipponbare; C403, mir156abcdfghikl. Ratio ≥ 1.5 or ≤ 0.75.

File Name: Supplementary Data 5
Description: Gene expression profiles in wild-type and mir156abcdfghikl seedling shoots. NIP, Nipponbare; D216, mir156abcdfghikl.

File Name: Supplementary Data 6
Description: Expression profiles of the GA biosynthetic, signaling and deactivating genes in wild-type and mir156abcdfghikl seedling shoots. NIP, Nipponbare; D216, mir156abcdfghikl.

File Name: Supplementary Data 7
Description: Expression profiles of the GA biosynthetic, signaling and deactivating DEGs in wild-type and mir156abcdfghikl seedling shoots. NIP, Nipponbare; D216, mir156abcdfghikl. Ratio ≥ 2 or ≤ 0.5.
