# Supplementary material for: The grain yield modulator miR156 regulates seed dormancy through the gibberellin pathway in rice
Source: Nat Commun. 2019 Aug 23;10:3822. doi: 10.1038/s41467-019-11830-5 (PMC6707268; doi:10.1038/s41467-019-11830-5)
Supplement: Supplementary file 11 — Reporting Summary [file 41467_2019_11830_MOESM11_ESM.pdf]

nature research

Corresponding author(s): Jian-Kang Zhu

Last updated by author(s): Jul 19, 2019

Reporting Summary

Nature Research wishes to improve the reproducibility of the work that we publish. This form provides structure for consistency and transparency in reporting. For further information on Nature Research policies, see Authors & Referees and the Editorial Policy Checklist.

Statistics

For all statistical analyses, confirm that the following items are present in the figure legend, table legend, main text, or Methods section.

n/a	Confirmed	
☐	☒	The exact sample size (*n*) for each experimental group/condition, given as a discrete number and unit of measurement
☐	☒	A statement on whether measurements were taken from distinct samples or whether the same sample was measured repeatedly
☐	☒	The statistical test(s) used AND whether they are one- or two-sided<br>*Only common tests should be described solely by name; describe more complex techniques in the Methods section.*
☐	☒	A description of all covariates tested
☐	☒	A description of any assumptions or corrections, such as tests of normality and adjustment for multiple comparisons
☐	☒	A full description of the statistical parameters including central tendency (e.g. means) or other basic estimates (e.g. regression coefficient) AND variation (e.g. standard deviation) or associated estimates of uncertainty (e.g. confidence intervals)
☐	☒	For null hypothesis testing, the test statistic (e.g. *F*, *t*, *r*) with confidence intervals, effect sizes, degrees of freedom and *P* value noted<br>*Give P values as exact values whenever suitable.*
☒	☐	For Bayesian analysis, information on the choice of priors and Markov chain Monte Carlo settings
☒	☐	For hierarchical and complex designs, identification of the appropriate level for tests and full reporting of outcomes
☒	☐	Estimates of effect sizes (e.g. Cohen's *d*, Pearson's *r*), indicating how they were calculated

Our web collection on statistics for biologists contains articles on many of the points above.

Software and code

Policy information about availability of computer code

Data collection	For RT-qPCR analyses, CFX Manager Software (Version 3.1, Bio-Rad) was used. For miR156 northern blots imaging, TImageQuent TL 7.0 was used for assessing the signal on phosphor screen.
Data analysis	We used Microsoft Excel 2016 and IBM SPSS Statistics 19 to analyze the data.

For manuscripts utilizing custom algorithms or software that are central to the research but not yet described in published literature, software must be made available to editors/reviewers. We strongly encourage code deposition in a community repository (e.g. GitHub). See the Nature Research guidelines for submitting code & software for further information.

Data

Policy information about availability of data

All manuscripts must include a data availability statement. This statement should provide the following information, where applicable:

- Accession codes, unique identifiers, or web links for publicly available datasets
- A list of figures that have associated raw data
- A description of any restrictions on data availability

The source data underlying all reported all averages in chart and tables, and uncropped versions of blots are provided as a Source Data file. The RNA-seq data generated in this study were deposited in the NCBI's Gene Expression Omnibus (GEO) Database under the accession number GSE131243, and the results of the RNA-seq analyses are available in Supplementary Data 2–7.

Field-specific reporting

Please select the one below that is the best fit for your research. If you are not sure, read the appropriate sections before making your selection.

☒ Life sciences ☐ Behavioural & social sciences ☐ Ecological, evolutionary & environmental sciences

For a reference copy of the document with all sections, see nature.com/documents/nr-reporting-summary-flat.pdf

Life sciences study design

All studies must disclose on these points even when the disclosure is negative.

Sample size	Sample size was determined based on previous studies and experience. We believe that our sample size is big enough to make our results powerful.
Data exclusions	No data were excluded from the analyses.
Replication	All experiments were conducted three or more times. The replications gave similar results.
Randomization	Plants were picked randomly for data collection.
Blinding	Before the studies, we were blinded to group allocation.

Reporting for specific materials, systems and methods

We require information from authors about some types of materials, experimental systems and methods used in many studies. Here, indicate whether each material, system or method listed is relevant to your study. If you are not sure if a list item applies to your research, read the appropriate section before selecting a response.

Materials & experimental systems

n/a	Involved in the study	
☐	☒	Antibodies
☒	☐	Eukaryotic cell lines
☒	☐	Palaeontology
☒	☐	Animals and other organisms
☒	☐	Human research participants
☒	☐	Clinical data

Methods

n/a	Involved in the study	
☒	☐	ChIP-seq
☒	☐	Flow cytometry
☒	☐	MRI-based neuroimaging

Antibodies

Antibodies used	We used anti-FLAG antibody (SIGMA. Catalog Number, F1084-5MG; clone, M2; Lot Number, SLBV9325) for the ChIP-qPCR assays. Immunodetection of SLR1 was performed using an anti-SLR1 primary antibody (1:2500) and an anti-IgG-HRP secondary antibody (Abmart, China). The anti-SLR1 primary antibody was kindly provided by Dr. Donglei Yang.
Validation	The anti-FlAG antibody and anti-IgG-HRP secondary antibody are commercial and specific. The anti-SLR1 primary antibody has been used in a recently published work by Dr. Donglei Yang's lab (Liu et al, 2019, Nature Plants 5, 389-400).
